# Vitamin D Deficiency as a Risk Factor for Diabetic Retinopathy: A Systematic Review and Meta-Analysis

**DOI:** 10.3390/biomedicines13010068

**Published:** 2024-12-30

**Authors:** Claudia Elena Petrea, Laura Andreea Ghenciu, Roxana Iacob, Emil Robert Stoicescu, Dorel Săndesc

**Affiliations:** 1 Department of Anatomy and Embriology, ‘Victor Babes’ University of Medicine and Pharmacy Timisoara, 300041 Timisoara, Romania; claudia.petrea@umft.ro (C.E.P.); roxana.iacob@umft.ro (R.I.); 2 Department of Functional Sciences, ‘Victor Babes’ University of Medicine and Pharmacy Timisoara, Eftimie Murgu Square No. 2, 300041 Timisoara, Romania; 3 Center for Translational Research and Systems Medicine, “Victor Babes” University of Medicine and Pharmacy Timisoara, Eftimie Murgu Square No. 2, 300041 Timisoara, Romania; 4 Doctoral School, “Victor Babes” University of Medicine and Pharmacy Timisoara, Eftimie Murgu Square No. 2, 300041 Timisoara, Romania; 5 Radiology and Medical Imaging University Clinic, “Victor Babes” University of Medicine and Pharmacy Timisoara, Eftimie Murgu Square No. 2, 300041 Timisoara, Romania; stoicescu.emil@umft.ro; 6 Research Center for Pharmaco-Toxicological Evaluations, “Victor Babes” University of Medicine and Pharmacy Timisoara, Eftimie Murgu Square No. 2, 300041 Timisoara, Romania; 7 Field of Applied Engineering Sciences, Specialization Statistical Methods and Techniques in Health and Clinical Research, Faculty of Mechanics, “Politehnica” University Timisoara, Mihai Viteazul Boulevard No. 1, 300222 Timisoara, Romania; 8 Department of Anesthesia and Intensive Care, “Victor Babes” University of Medicine and Pharmacy Timisoara, Eftimie Murgu Square 2, 300041 Timisoara, Romania; sandesc.dorel@umft.ro

**Keywords:** diabetes mellitus, diabetic retinopathy, vitamin D, vitamin D deficiency, 25-hydroxyvitamin D, systematic review, meta-analysis

## Abstract

Diabetic retinopathy (DR), a significant microvascular complication of diabetes mellitus (DM), remains a major cause of vision loss worldwide. Vitamin D, recognized for its role in bone health, has also been implicated in various non-skeletal conditions, including DR. This systematic review analyzed data from 20 studies involving 22,408 participants to explore the relationship between vitamin D levels and DR. Studies were included based on strict eligibility criteria, ensuring they could distinctly classify participants into DR and non-DR groups and provide quantitative measurements of vitamin D levels. Of these, nine studies were included in the meta-analysis. The pooled analysis revealed a significant association between lower vitamin D levels and increased odds of DR, with a combined odds ratio (OR) of 1.15 (95% CI: 1.10–1.20) under the fixed-effects model and 1.17 (95% CI: 1.08–1.27) under the random-effects model. Mean serum vitamin D levels were lower in individuals with DR (18.11 ± 5.35 ng/mL) compared to those without DR (19.71 ± 7.44 ng/mL), with a progressive decline observed across DR severity stages. Subgroup analyses showed significantly lower levels of vitamin D in proliferative DR compared to non-proliferative stages. Heterogeneity (I^2^ = 89%) was noted, most probably due to geographic differences, varying methodologies for vitamin D measurement, and DR classification approaches. Secondary analyses indicated that vitamin D deficiency prevalence ranged from 27% to 95% in DR populations, highlighting its potential role in disease progression. This review highlights the need for longitudinal studies to better understand the causal relationship. The findings also call attention to a critical gap in the literature regarding the therapeutic role of vitamin D supplementation in preventing and managing DR. Addressing vitamin D deficiency as a modifiable risk factor in DM care may offer new avenues for reducing the burden of DR.

## 1. Introduction

Diabetes mellitus (DM) is a chronic metabolic disorder characterized by impaired glucose regulation, which can lead to severe complications affecting multiple organ systems. As a major public health concern, DM has reached epidemic proportions, affecting more than 300 million individuals worldwide, with rising prevalence driven by aging populations, urbanization, unhealthy lifestyles, and obesity [1]. The condition is associated with significant morbidity and mortality, with poorly controlled blood glucose placing patients at high risk for macrovascular and microvascular complications, including cardiovascular disease, diabetic retinopathy (DR), and nephropathy. These complications often result in progressive organ dysfunction and reduced quality of life. Its impact extends beyond individual health, imposing substantial economic burdens on healthcare systems due to the costs of managing chronic complications [1,2,3].

DR is a leading cause of vision impairment and blindness among individuals with DM worldwide [4]. As a microvascular complication, DR results from chronic hyperglycemia-induced damage to retinal blood vessels, leading to increased vascular permeability, ischemia, and progressive retinal injury. Metabolic control, blood pressure, chronic inflammation and the duration of diabetes are well-established independent risk factors for DR [5,6]. However, the precise pathophysiological mechanisms contributing to this condition are not yet fully understood.

Vitamin D, a secosteroid hormone, primarily obtained through sun exposure and dietary intake, has gained attention for its potential role in maintaining vascular and retinal health. Beyond its traditional role in calcium homeostasis and bone metabolism, vitamin D exerts anti-inflammatory, immunomodulatory, and anti-angiogenic effects, which are crucial for preserving the integrity of the retinal vasculature [7,8]. Low vitamin D levels have been implicated in several chronic conditions, including cardiovascular disease, DM, and hypertension, which are also recognized risk factors for DR [7,8]. Vitamin D deficiency is highly prevalent among patients with type 2 DM [9]. It plays a crucial role in retinal health, with its receptors widely expressed in the retina, influencing processes such as inflammation, angiogenesis, and neuroprotection [10,11]. The integrity of retinal endothelial cells is critical for maintaining the retinal blood barrier and proper vision, and, while vitamin D, through its nuclear receptor (VDR), plays a protective role in EC function by regulating genes involved in angiogenesis and inflammation, its impact on retinal EC function and ocular angiogenesis remains underexplored [12].

Emerging evidence suggests an association between vitamin D deficiency and the development or severity of DR; however, results across studies remain inconsistent, with some reporting no significant association [9]. These discrepancies may be attributed to variations in study designs, populations, and methods used to measure vitamin D levels and DR severity [13].

The objective of this systematic review and meta-analysis is to synthesize the current evidence on the relationship between vitamin D levels and DR. By pooling data from existing studies, we aim to determine whether low vitamin D levels are associated with an increased risk of DR and explore potential differences based on the severity of DR. This work seeks to provide a clearer understanding of the role of vitamin D in DR pathogenesis and inform future research and clinical strategies targeting modifiable risk factors for this vision-threatening condition.

## 2. Materials and Methods

This systematic review and meta-analysis were conducted following the Preferred Reporting Items for Systematic Reviews and Meta-Analyses (PRISMA) guidelines [14]. The aim was to evaluate the relationship between serum vitamin D levels and DR.

### 2.1. Data Search Strategy

A comprehensive and systematic literature search was performed across multiple electronic databases, including PubMed, Google Scholar, and Scopus, to identify relevant studies published up to October 2024. A combination of controlled vocabulary (MeSH terms in PubMed) and free-text keywords was employed to capture all potential studies related to vitamin D and diabetic retinopathy. The primary search terms included “vitamin D”, “25-hydroxyvitamin D”, “diabetic retinopathy”, “diabetes”, “diabetes mellitus”, “vitamin D deficiency”, and “retinal complications”. Boolean operators (AND/OR) were applied to combine these terms effectively. To refine the search, filters were applied where possible, including limiting studies to human subjects, full-text availability, and peer-reviewed publications. No restrictions were imposed on study design or geographic location, to ensure inclusivity. In addition to the primary database searches, the reference lists of all included studies, relevant review articles, and meta-analyses were manually screened to identify additional eligible studies that may have been missed in the initial search. Grey literature, such as unpublished theses or conference abstracts, was not included, to ensure the methodological rigor of the review.

### 2.2. Eligibility Criteria

The dataset for this systematic review and meta-analysis was curated to address the aim of exploring the relationship between vitamin D levels and DR. Studies were included based on their ability to provide comparisons between groups with and without DR. We included observational designs (cross-sectional, case-control, and cohort studies) and randomized controlled trials (RCTs) that reported serum 25-hydroxyvitamin D [25(OH)D] levels. All included studies defined DR status through assessments performed by ophthalmologists or using standardized DR grading systems., involved patients with type 1 DM, type 2 DM, or both, and defined vitamin D deficiency based on specific thresholds. All included studies featured patients with low levels of vitamin D. The primary outcomes of the studies were to compare the presence or severity of diabetic retinopathy in patients with or without low levels of vitamin D or to assess differences in vitamin D levels between patients with DR and controls. Additional inclusion and exclusion criteria are listed in Table 1. The PICO (Population-Intervention-Comparison-Outcome) framework provided a structured approach to this study, guiding the identification and selection of relevant research and ensuring a focused analysis of the association between vitamin D levels and DR (Table 2). To ensure comprehensive coverage, the researchers also hand-searched relevant articles and references to capture all potentially eligible studies.

The meta-analysis included studies that reported quantitative data on vitamin D levels for individuals with DR and those without DR. To be eligible, studies had to provide mean and standard deviation values or data sufficient to calculate them for both groups. Additionally, studies were required to include groups of patients with and without DR, assessed either by ophthalmologists or through standardized guidelines. Additionally, studies needed to be peer-reviewed and provide data in a format suitable for statistical pooling. Studies without this data or with unclear group definitions were excluded from the meta-analysis.

### 2.3. Data Extraction and Management

Data extraction was performed independently by two reviewers using a standardized form. Extracted information included study characteristics (authors, year, location, design, and sample size), participant demographics (age, gender, diabetes type), serum vitamin D levels (mean, standard deviation, units, and cutoff values), and DR classification (severity). Any discrepancies between reviewers were resolved through discussion or consultation with a third reviewer. Data extraction was verified for completeness and accuracy before proceeding with analysis.

### 2.4. Meta-Analysis and Statistical Approach

The primary outcome of this systematic review and meta-analysis was to evaluate the association between serum vitamin D levels and DR, with effect sizes expressed as pooled odds ratios (ORs) with 95% confidence intervals (CIs). A meta-analysis was conducted primarily using a random-effects model to account for variability between studies. Sensitivity analysis was performed using a fixed-effects model to assess the consistency of the findings. Heterogeneity among studies was assessed using the Q-statistic and I^2^ statistic. Secondary outcomes included the calculation of mean serum vitamin D levels and the prevalence of vitamin D deficiency in individuals with and without DR. Subgroup analyses were performed to examine differences based on DR severity (non-proliferative, pre-proliferative, and proliferative DR stages, where available). Risk of bias was evaluated using the Newcastle–Ottawa Scale (NOS) for case-control, cohort, and cross-sectional studies, while the Risk of Bias 2 (RoB 2) tool was applied for randomized controlled trials. Publication bias was examined through visual inspection of funnel plots and statistically assessed using Egger’s test, with *p*-values < 0.05 indicating significant asymmetry. All statistical analyses were performed using RevMan 5.4 (Cochrane Collaboration, London, UK).

### 2.5. Data Visualization

Results are presented using tables and visual displays (boxplot, funnel plot). A summary table included study characteristics. Forest plots showed pooled odds ratios and confidence intervals for the meta-analysis. Funnel plots and Egger’s test assessed publication bias, and the PRISMA flowchart detailed the study selection process. Risk-of-bias assessments were tabulated using the Newcastle–Ottawa Scale and RoB 2 tool.

### 2.6. Protocol Development and Ethical Considerations

This systematic review and meta-analysis protocol were developed in accordance with the PRISMA (Preferred Reporting Items for Systematic Reviews and Meta-Analyses Protocols) guidelines [14]. The completed PRISMA checklist is provided as Supplementary Materials for reference (Table S1). The protocol was registered with PROSPERO, the international prospective register of systematic reviews (CRD42024615053). As the systematic review does not involve patient recruitment or the collection of personal information, approval from an ethics committee was not required.

## 3. Results

**Figure 1:**
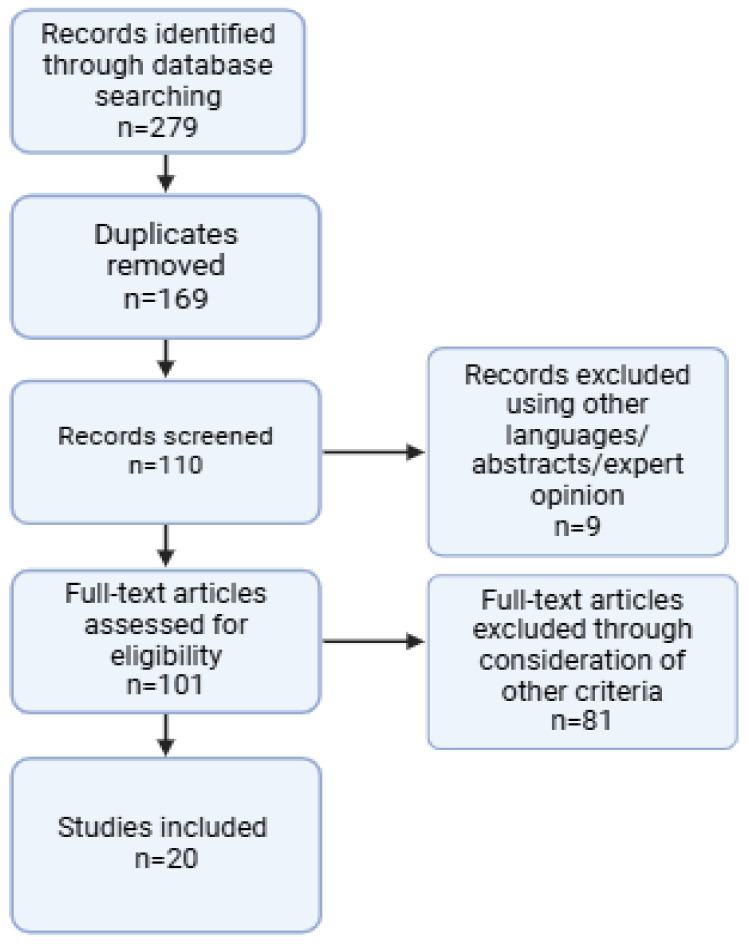
PRISMA flowchart. Created with Biorender.

After screening the articles from the initial database search, 20 studies comprising a total of 22,408 participants met the inclusion criteria for this systematic review, following a rigorous selection process and PRISMA guidelines (Figure 1) [8,13,15,16,17,18,19,20,21,22,23,24,25,26,27,28,29,30,31,32].

### 3.1. Demographic Analysis

The details and characteristics of the studies included in this meta-analysis are presented in Table 3 [8,13,15,16,17,18,19,20,21,22,23,24,25,26,27,28,29,30,31,32]. The studies included were identified through our systematic search strategy and cross-referenced with the original articles. Studies from Europe were conducted in Spain (two studies, 668 participants), Italy (one study, 715 participants), Portugal (one study, 182 participants), and the UK (one study, 657 participants), reflecting populations with varying dietary habits and sunlight exposure. In Asia, large studies were performed in Iran (two studies, 637 participants), Turkey (one study, 557 participants), Tibet (one study, 365 participants), China (two studies, 3886 participants), Bahrain (one study, 300 participants), South Korea (one study, 65 participants), and India (three studies, 3290 participants). From America, two studies in the United States included 842 and 1339 participants, and one study in French Guinea included 361 participants. Meanwhile, Oceania and Northern Europe were represented by a combined study from Australia, New Zealand, and Finland involving 9524 participants. The review included four case-control studies, 10 cross-sectional studies, three retrospective observational studies, two prospective observational studies, and one randomized-controlled study. Vitamin D levels were measured using various methods across the studies, including chemiluminescence assays (10 studies), high-performance liquid chromatography (two studies), radioimmunoassay (two studies), and high-sensitivity mass spectrometry (two studies). The vast majority of included studies relied on ophthalmologists to objectively diagnose DR. However, two studies used the modified Airlie House classification system, which provides a standardized, systematic approach for grading DR severity.

### 3.2. Primary Analysis

#### 3.2.1. Comparative Vitamin D Levels in Diabetic Retinopathy and Non-Diabetic Retinopathy Groups

**Figure 2:**
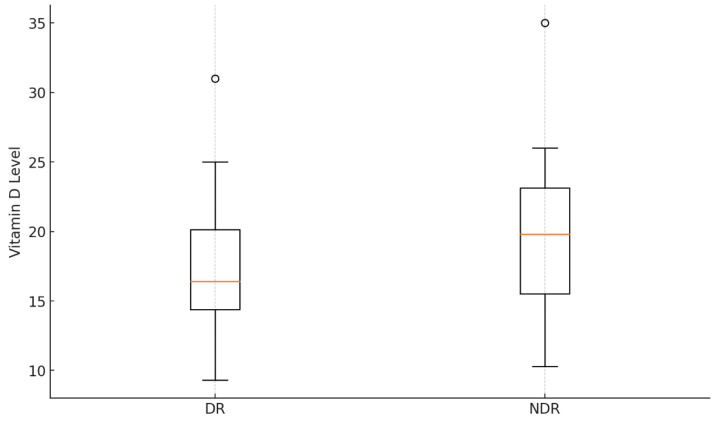
Boxplot comparing serum vitamin D levels between patients with DR and those without DR.

Among the 20 studies analyzed, 13 provided data comparing vitamin D levels between individuals with DR and those without DR (Figure 2). One study lacked sufficient data to allow a direct comparison of vitamin D levels between DR and non-DR groups, and six other studies did not provide relevant data for this analysis. Additionally, four studies analyzed vitamin D levels across different stages of DR.

Most studies showed that mean vitamin D levels in the DR group were lower than those in the non-DR (NDR) group. For instance, in one study, the mean vitamin D level was 19.2 ± 10.1 ng/mL in DR compared to 20.5 ± 8.1 ng/mL in NDR, showing a modest difference [15]. Similarly, in another study, individuals with DR had a mean level of 9.2 ± 7.0 ng/mL compared to 10.3 ± 9.4 ng/mL in NDR [18], indicating a slight but consistent trend. Notably, more significant differences were observed in some studies, such as a mean vitamin D level of 14.46 ng/mL in DR versus 19.88 ng/mL in NDR [30], and 14.10 ± 1.20 ng/mL in DR versus 23.30 ± 2.01 ng/mL in NDR [26].

However, not all studies found substantial differences between the DR and NDR groups. In some cases, the mean levels were relatively similar, such as 16.4 ± 10.5 ng/mL in DR and 15.3 ± 9.0 ng/mL in NDR [13], reflecting the potential influence of confounding factors such as glycemic control or duration of diabetes.

The analysis revealed that the mean Vitamin D level in the DR group was 18.11 ng/mL with a standard deviation of 5.35 ng/mL, while the NDR group had a slightly higher mean of 19.71 ng/mL and a standard deviation of 7.44 ng/mL. The distributions indicate some overlap, with the NDR group showing greater variability compared to the DR group.

Geographic differences in vitamin D levels were prominent, with studies from Asia (India, China, Iran, and Turkey) consistently reporting lower levels compared to those from Europe and North America, likely due to differences in sunlight exposure, lifestyle and diet. Bonakdaran et al. [18] reported mean levels of 9.2 ± 7.0 ng/mL in DR patients versus 10.3 ± 9.4 ng/mL in non-DR patients. Similarly, Nadri et al. [26] observed levels of 14.10 ± 1.20 ng/mL in DR, compared to 23.30 ± 2.01 ng/mL in NDR patients. Girard et al. [21] in French Guinea reported notably higher mean vitamin D levels (31 ng/mL) compared to other regions, suggesting environmental or population-specific factors at play.

Overall, the trend of lower mean vitamin D levels in DR compared to NDR suggests a possible association, reinforcing the hypothesis that vitamin D deficiency may play a role in DR development or progression.

#### 3.2.2. Vitamin D Levels Across Stages of Diabetic Retinopathy

The analysis of 25(OH)D levels across the four datasets that reported these levels across different DR stages consistently demonstrates a trend of decreasing vitamin D levels as DR progresses from non-proliferative diabetic retinopathy (NPDR) to proliferative diabetic retinopathy (PDR). This trend is most evident in the studies of Alcubierre et al. [15] and Nadri et al. [26], where significant reductions in vitamin D levels are observed in the advanced stages of DR. Specifically, Alcubierre et al. demonstrate a statistically significant difference between NDR and PDR (*p* = 0.02), while Nadri et al. show highly significant differences across all stages of DR (*p* < 0.001), reinforcing the link between lower vitamin D levels and DR progression.

Although the studies of Bonakdaran [18] and Alam [13] do not show statistically significant differences, their findings align with the overall trend of declining 25(OH)D levels as DR severity increases. This consistency across datasets highlights the potential role of vitamin D deficiency in DR progression, even when statistical significance is not achieved in some cases. The vitamin D levels were observed to range from approximately 10.3 ng/mL to 23.3 ng/mL in NDR, decrease to between 9.0 ng/mL and 18.1 ng/mL in NPDR, and range between 10.1 ng/mL and 14.1 ng/mL in PDR.

The significant associations observed in two datasets provide a strong foundation for investigating whether vitamin D supplementation could play a role in the management or prevention of DR.

### 3.3. Secondary Analysis

The prevalence of vitamin D deficiency (VDD) varies significantly between individuals with DR and NDR, reflecting differences in populations, geographic locations, and study methodologies. Among individuals with DR, VDD prevalence ranges from 27% to as high as 95.33%, while in those with NDR, the prevalence ranges from 23% to 94%. In some studies, VDD prevalence was notably higher in DR compared to NDR. For instance, one study reported 61.9% VDD in DR compared to 50.7% in NDR [15], while another observed 71.6% VDD in DR and 67.5% in NDR [17]. However, this pattern was not consistent across all studies. In some cases, VDD prevalence was comparable between the groups, or even slightly higher in NDR. For example, one study reported 45.2% VDD in DR compared to 54.2% in NDR [28], while another found 29.6% in DR compared to 70.4% in NDR [29].

Overall, vitamin D deficiency prevalence was highest in Middle Eastern and South Asian populations, aligning with the lower reported mean levels. Additionally, variability in the methods used to assess vitamin D levels may have contributed to minor inconsistencies across studies.

### 3.4. Meta-Analysis

Out of 13 studies that met the preliminary criteria for potential inclusion in the meta-analysis, four were excluded, resulting in the inclusion of nine studies [17,19,20,22,23,24,26,28,32]. The exclusions were due to methodological limitations: no confidence intervals/standard errors or non-significant *p*-values. While this exclusion may introduce a minor risk of bias, the strict inclusion criteria ensured the reliability of the synthesized results.

**Figure 3:**
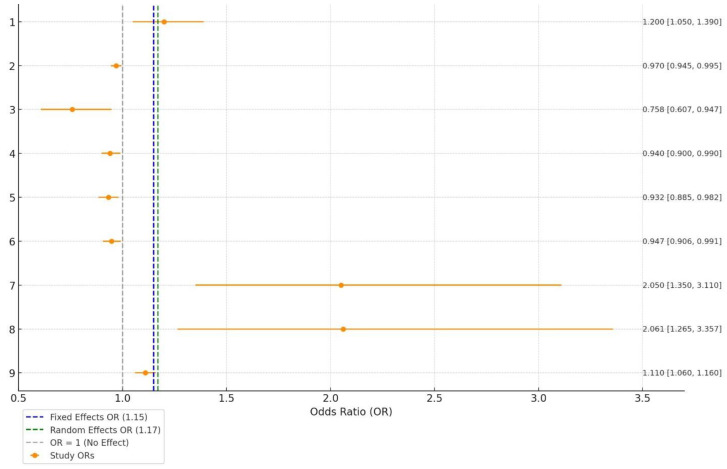
Forest plot depicting the odds ratios for the association between serum vitamin D levels and DR across multiple studies.

The meta-analysis revealed a significant association between vitamin D deficiency and DR (Figure 3). The random-effects model, accounting for potential variability among the studies, yielded a combined OR of 1.17 with a 95% CI of (1.08, 1.27). Using the fixed-effects model, the combined OR was calculated as 1.15 with a 95% CI of (1.10, 1.20). This indicates a consistent relationship across studies, suggesting that individuals with lower vitamin D levels have a higher likelihood of developing DR.

We assessed heterogeneity among the included studies, calculating both the Q-statistic and the I^2^ statistic. In this analysis, the Q-statistic was 72.75, indicating variability across studies. The calculated I^2^ value was 89.00%, signifying considerable heterogeneity. This high level of heterogeneity suggests substantial differences between the studies, possibly due to variations in study design, population characteristics, or other methodological factors. Geographically, studies were conducted across Europe, Asia, America, and Oceania, regions with varying levels of sunlight exposure, dietary vitamin D intake, and healthcare practices. Additionally, diverse methodologies for assessing serum vitamin D levels, such as chemiluminescence, high-performance liquid chromatography, radioimmunoassay, and high-sensitivity mass spectrometry, may introduce variability. The approaches for diagnosing and classifying DR also varied, with most studies relying on ophthalmological assessment, while two studies employed the standardized modified Airlie House classification system.

Overall, both models highlight that vitamin D deficiency is a notable risk factor for DR, emphasizing the potential importance of monitoring and addressing vitamin D levels in individuals with DM.

**Figure 4:**
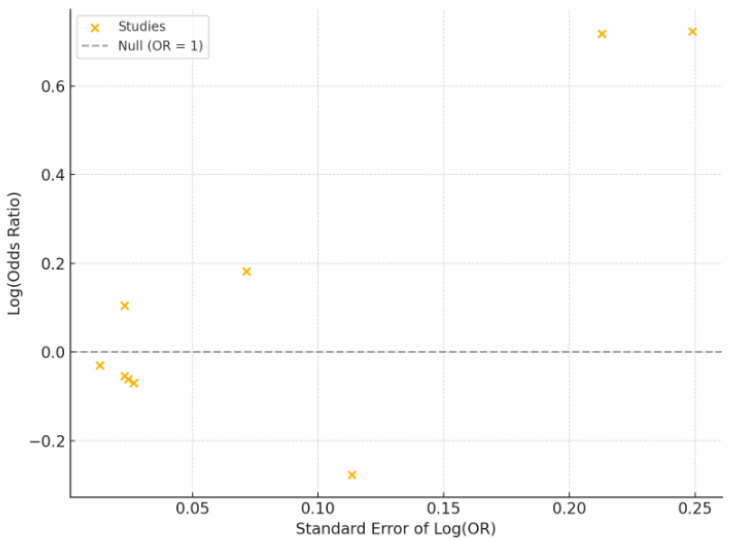
Funnel plot assessing publication bias and heterogeneity among studies.

The funnel plot showed a relatively symmetric distribution of studies around the central line (OR = 1), suggesting no strong visual evidence of publication bias (Figure 4). This conclusion was further supported by Egger’s test, which yielded a p-value of 0.156, indicating no statistically significant asymmetry in the plot.

### 3.5. Study Bias

We evaluated the risk of bias for all 20 included studies, with a summary of the assessments presented in Table 4, Table 5 and Table 6. Among these, nine studies met the criteria for inclusion in the meta-analysis, selected based on the availability of relevant data and the comparability of reported outcomes. The risk-of-bias assessments were conducted using the Newcastle–Ottawa Scale for case-control, cross-sectional, and cohort studies. Out of the 19 studies, three were classified as having low risk of bias, 10 had a low-to-moderate risk, and six had a moderate risk. All studies met the inclusion criteria for quality assessment, as the overall risk of bias remained within acceptable limits. Studies with a higher risk of bias would have been excluded, to ensure the reliability of our findings. The study by Hermann, assessed as a randomized controlled trial using the Cochrane Risk of Bias (RoB) 2 tool, demonstrated a low risk of bias across all domains, including random sequence generation, allocation concealment, baseline imbalance, blinding, adherence, completeness of data, reasons for missing data, blinding of outcome assessors, use of objective outcome measures, and reporting completeness, indicating high methodological quality overall.

## 4. Discussion

In this systematic review and meta-analysis, we aimed to evaluate the relationship between serum vitamin D levels and the risk of DR by synthesizing data from observational and RCT studies across diverse populations, with a specific focus on highlighting low vitamin D levels in conjunction with the presence of DR, its severity, and the mean vitamin D levels across different groups. The dataset included in this review was selected to provide a balanced understanding of the association between vitamin D deficiency and DR. The studies represent diverse populations, methodological approaches, and vitamin D measurement techniques, allowing for generalizability of findings. The pooled results demonstrated a significant association between vitamin D deficiency and DR, with a fixed-effects model yielding a combined OR of 1.15 (95% CI: 1.10–1.20) and a random-effects model confirming the association with an OR of 1.17 (95% CI: 1.08–1.27). Furthermore, subgroup analyses revealed consistently lower mean serum vitamin D levels in patients with DR compared to those without, with levels declining progressively across stages of DR severity (non-DR, non-proliferative DR, and proliferative DR).

The results of this meta-analysis may have been influenced by several factors across the included studies. First, variations in study design, such as cross-sectional versus longitudinal approaches, could impact the strength and direction of the observed associations. We included 10 cross-sectional studies, four case-control studies, three retrospective studies, two prospective studies and one RCT in this systematic review. Second, differences in the methods used to measure serum vitamin D levels and DR severity might introduce measurement bias, as some studies employed different assays or diagnostic criteria for DR classification. Across studies, vitamin D level was assessed using chemiluminescence (10 studies), high performance liquid chromatography (two studies), radioimmunoassay (two studies), and high-sensitivity mass spectrometry (two studies). Geographic and environmental factors, such as sunlight exposure and dietary vitamin D intake, also likely contributed to variability in baseline vitamin D levels between populations. Additionally, confounding factors like glycemic control, diabetes duration, comorbidities, and the use of vitamin D supplements were inconsistently reported or controlled, which may have affected the accuracy of the observed associations. Lastly, differences in sample size and statistical power across studies could result in varying levels of precision, influencing the pooled estimates.

DR stands as the most prevalent ocular complication associated with diabetes and represents a leading cause of blindness worldwide, particularly in working-age populations [33]. Its progression is marked by a series of pathophysiological changes that reflect both microvascular damage and inflammatory responses within the retina. In the early stages, thickening of the capillary endothelial basement membrane, pericyte apoptosis, and the disruption of capillary autoregulation collectively compromise microvascular integrity [34,35]. These alterations result in endothelial barrier dysfunction and the breakdown of the blood–retina barrier, allowing blood components such as proteins and lipids to leak into retinal tissues. This leads to the formation of retinal edema, a hallmark feature of early DR. Capillary occlusion further exacerbates ischemia, setting the stage for progressive retinal damage [30].

As DR advances, the retina responds to ischemia by inducing pathological neovascularization and angiogenesis, characterized by the growth of fragile, abnormal blood vessels. These vessels are prone to rupture, causing vitreous hemorrhages and exacerbating retinal injury. In addition, fibrotic hyperplasia may occur, contributing to retinal scarring and, in severe cases, tractional retinal detachment, which significantly impairs vision [35]. The transition from non-proliferative to proliferative DR marks a critical juncture in disease progression, often requiring more aggressive therapeutic interventions [36].

The pathogenesis of DR is complex and multifactorial, with hyperglycemia being a central driver. Chronic hyperglycemia induces oxidative stress, promotes the adhesion of leukocytes to retinal microvascular endothelial cells, and triggers an inflammatory cascade that damages endothelial cells and pericytes [37,38,39]. These processes result in altered hemorheology, increased vascular permeability, and eventual microvascular occlusion [35]. Moreover, advanced glycation end products (AGEs), produced under hyperglycemic conditions, further contribute to microvascular damage and inflammation, amplifying retinal destruction [40].

Emerging evidence also suggests a significant role for systemic factors, such as hypertension, dyslipidemia, and vitamin D deficiency, in the progression of DR [41]. These factors exacerbate the underlying microvascular and inflammatory mechanisms, highlighting the importance of comprehensive metabolic control in managing DR risk [35,42]. Furthermore, recent studies have focused on the contribution of neurovascular interactions, emphasizing that DR is not solely a vascular disease but also involves significant neurodegenerative components, such as retinal ganglion cell loss and glial dysfunction [43,44].

Vitamin D insufficiency and deficiency are highly prevalent in the general population [45]. Beyond its established role in bone metabolism, vitamin D exhibits a wide range of pleiotropic effects [46]. Vitamin D undergoes activation through two hydroxylation steps in the liver and kidneys, producing the active form 1,25(OH)2D, which exerts genomic actions by regulating gene expression via nuclear VDR and rapid nongenomic effects, potentially mediated by a membrane receptor, while its status is primarily assessed through 25(OH)D levels due to its longer half-life and stability [47]. Preclinical studies suggest that vitamin D can influence β-cell growth and differentiation, promote insulin secretion [48], upregulate insulin receptor expression [49], and improve insulin-mediated glucose transport [50]. Vitamin D has also been shown to inhibit VEGF-induced endothelial cell sprouting, elongation, and proliferation, as demonstrated in a mouse model by Albert et al. [26,51]. Another study concluded that vitamin D shows its protective effect on DR through multiple mechanisms, including preserving retinal structure and thickness, reducing vascular permeability, and inhibiting retinal cell apoptosis. This is achieved by downregulating the TXNIP/NLRP3 inflammasome pathway, decreasing reactive oxygen species production, and modulating the Bax/Bcl-2 ratio to suppress mitochondrial stress and apoptosis [52]. Vitamin D also suppresses pro-inflammatory cytokines such as tumor necrosis factor (TNF)-α, interleukin (IL)-6, and IL-1β while promoting anti-inflammatory mediators like IL-10 [53]. Numerous studies indicate an inverse correlation between vitamin D levels and DR; however, the causal relationship remains unclear. It is uncertain whether low vitamin D levels contribute to the development of DR or if DR leads to reduced vitamin D levels due to factors such as sedentarism and limited sun exposure [41]. While VDD has been linked to various adverse health outcomes, vitamin D supplementation offers a low-cost and safe therapeutic approach, making it an appealing option for both clinicians and researchers.

Genetic variants of vitamin D-related genes, particularly those affecting the VDR, have been increasingly studied for their potential role in the development of DR. Polymorphisms such as Bsm1 (rs1544410) [54], Fok1 (rs2228570) [55], and Taq1 (rs731236) [56] in the VDR gene have been associated with varying risks of DR in different populations. These variants can influence the transcriptional activity of the VDR gene and its downstream signaling pathways, which regulate key processes in DR pathogenesis. Hong et al. demonstrated that Korean patients with type 2 diabetes who carried the B allele (BB or Bb) of the Bsm1 polymorphism in the VDR gene had a lower risk of developing diabetic retinopathy compared to those without the B allele (bb) [54], which suggests that the presence of B allele might confer protection by modulating the anti-inflammatory and antioxidant properties of vitamin D. Longo-Mbenza et al. reported that heightened oxidative stress biomarkers and reduced antioxidant levels are significantly linked to the earlier onset of DR in African individuals with type 2 DM, suggesting that vitamin D supplementation could be recommended as a complementary therapy for managing this disease [57]. In another study, genetic analysis showed no significant differences in serum 25(OH)D across VDR genotypes, but TT of rs1544410 or GG of rs731236 increased the risk of microvascular complications of DM by 23%, while 25(OH)D ≥50 nmol/L and major allele homozygotes reduced this risk significantly, suggesting a protective threshold of vitamin D levels that could counteract genetic vulnerability [58]. These genetic variations may influence the expression and functionality of VDR, thereby modulating vitamin D’s role in regulating inflammation, angiogenesis, and oxidative stress.

Supplementation with vitamin D has emerged as a potential therapeutic approach to limit the progression of DR, particularly in individuals with vitamin D deficiency. Evidence suggests that correcting VD levels through supplementation may enhance retinal health by reducing inflammation, oxidative stress, and apoptosis of retinal cells, all of which contribute to DR pathogenesis [59].

A recent meta-analysis of randomized controlled trials demonstrated that vitamin D supplementation in patients with type 2 DM can lead to improvements in HbA1c, insulin resistance, and insulin secretion during short-term interventions [60]. Additionally, another meta-analysis of studies revealed that vitamin D administration had a favorable effect on fasting glucose levels, specifically in patients with poorly controlled diabetes [61]. In another study, vitamin D supplementation (50,000 IU weekly for 8 weeks, followed by 800 IU daily) was given to diabetic macular edema (DME) patients with vitamin D insufficiency (10–30 ng/mL) alongside intravitreal bevacizumab. Results showed that vitamin D correction significantly improved visual acuity and reduced central macular thickness at 6 months compared to bevacizumab alone, particularly after the third month, suggesting its potential role as an adjunct therapy in DME management [62]. Additionally, studies indicate that the co-administration of magnesium with vitamin D may optimize its bioavailability and effectiveness, as magnesium acts as a critical cofactor in converting vitamin D into its active form [63]. Studies have demonstrated that combined magnesium and vitamin D supplementation significantly increases serum 25-hydroxyvitamin D levels, particularly in individuals with vitamin D insufficiency or obesity [64]. This synergistic effect shows the importance of addressing both vitamin D and magnesium deficiencies when considering supplementation strategies for improving retinal outcomes in patients with diabetes.

This systematic review and meta-analysis highlight the significant association between vitamin D deficiency and DR, yet several gaps remain that warrant further investigation. Future research should prioritize longitudinal and interventional studies to establish causality and clarify whether vitamin D deficiency contributes directly to DR development or if DR leads to reduced vitamin D levels. Standardizing methods for measuring serum 25-hydroxyvitamin D and DR classification is essential to improve comparability across studies. Additionally, further exploration of the molecular mechanisms underlying vitamin D’s anti-inflammatory, anti-angiogenic, and neuroprotective effects on retinal health is needed. The role of genetic variations in the VDR and their influence on DR susceptibility should be investigated more comprehensively, particularly in diverse populations. Research should also focus on subgroup analyses to understand the impact of vitamin D levels across different diabetes types, ethnicities, geographic regions, and stages of DR severity. Randomized controlled trials are required to evaluate the efficacy of vitamin D supplementation in preventing or slowing DR progression and to determine optimal dosing strategies. Lastly, future studies must address potential confounding factors, such as glycemic control, diabetes duration, comorbidities, and lifestyle influences, to ensure more robust and accurate findings.

## 5. Conclusions

This systematic review and meta-analysis provide evidence of a significant association between vitamin D deficiency and diabetic retinopathy. Patients with lower serum vitamin D levels exhibited higher odds of developing DR, with progressively lower levels observed across stages of DR severity. The pooled results across multiple studies showed that individuals with sufficient vitamin D levels had a reduced risk of DR compared to those with deficient levels. These findings emphasize the potential role of vitamin D as a protective factor in managing DR and warrant further research to validate its therapeutic implications.

## Figures and Tables

**Table 1:** Inclusion and exclusion criteria.

Inclusion Criteria	Exclusion Criteria
Observational studies (cross-sectional, case-control, cohort, propective/retrospective)	Reviews, editorials, abstracts, expert opinions, or case reports
Evaluated 25(OH)D levels and diabetic retinopathy	Lacked control group
Standardized laboratory measurements of 25(OH)D	Non-standardized or missing vitamin D measurements
Participants with type 1 or type 2 diabetes	Non-English or inaccessible full-text studies.
At least one group of patients with low levels of vitamin D	
English language	
Published in the last 10 years	

**Table 2:** PICO framework.

Population	Individuals with type 1 or type 2 diabetes, with or without diabetic retinopathy
Intervention	Assessment of serum 25-hydroxyvitamin D levels
Comparison	Primary analysis: comparison of vitamin D levels between individuals with and without DR Secondary analysis: comparison of vitamin D levels between stages of DR
Outcome	Association between serum 25(OH)D levels and DR, expressed as odds ratios, relative risks, or mean differences.

**Table 3:** Clinical and demographic characteristics of the included studies.

Author, Year	Country	Study Design	Population	Vitamin D Assesement	DR Assesement	Mean 25-OH DR	Mean 25-OH No DR	DM
Alam et al., 2016 [13]	UK	CSS	657	CL	Ophtalmologist	16.4 ± 10.5 15.9 ± 10.4 15.7 ± 8.5	15.3 ± 9.0	T1/T2
Alcubierre et al., 2015 [15]	Spain	CC	283	CL	Ophtalmologist	19.2 ± 10.1	20.5 ± 8.1	T2
Almoosa et al., 2019 [16]	Bahrain	CSS	300	N/A	Ophtalmologist	12.6	N/A	T2
Ashinne et al., 2018 [17]	India	Retrospective	3054	ECL	Ophtalmologist	11.9 ± 2.2	13.7 ± 2.1	T2
Bonakdaran et al., 2015 [18]	Iran	CSS	235	RIA	Ophtalmologist	9.2 ± 7.0	10.3 ± 9.4	T2
Castillo-oti et al., 2021 [19]	Spain	CC	385	CL	Ophtalmologist	24.5	35	T2
Chen et al., 2024 [20]	Tibet	CSS	365	CL	Ophtalmologist	9.3	11.3	T2
Girard et al., 2021 [21]	French Guinea	CSS	361	N/A	Ophtalmologist	31	26	N/A
Hermann et al., 2015 [22]	Australia	RCT	9524	CL	Ophtalmologist	N/A	N/A	T2
Kim et al., 2019 [8]	South Korea	Prospective	65	Immunoassay	Ophtalmologist	14.3 ± 9.1	16.2 ± 8.0	N/A
Long et al., 2017 [23]	USA	CSS	842	LC-MS	Ophtalmologist	N/A	N/A	N/A
Lopes et al., 2020 [24]	Portugal	Retrospective	182	N/A	Ophtalmologist	21 ± 9.5/ 20.3 ± 11.8/ 19.8 ± 10.8	22.6 ± 11.2	T1
Millen et al., 2016 [25]	USA	Prospective	1339	LC-MS	Modified Airlie House Classification	N/A	N/A	N/A
Nadri et al., 2019 [26]	India	CSS	72	CL	Ophtalmologist	18.10 ± 1.90/ 14.10 ± 1.20	23.30 ± 2.01	T2
Reddy et al., 2015 [27]	India	CC	164	HPLC	Modified Airlie House Classification	N/A	N/A	T2
Usluogullari et al., 2015 [28]	Turkey	Retrospective	557	HPLC	Ophtalmologist	20.0 ± 7.7	19.7 ± 8.4	T2
Xiao et al., 2020 [29]	China	CSS	3071	CL	Ophtalmologist	N/A	N/A	T2
Zahedi et al., 2024 [30]	Iran	CC	402	CL	Ophtalmologist	14.46	19.88	T2
Zhao et al., 2021 [31]	China	CSS	815	ECL	Ophtalmologist	16.38 ± 9.16	16.23 ± 7.10	T2
Zoppini et al., 2015 [32]	Italy	CSS	715	CL	Ophtalmologist	N/A	N/A	T2

Abbreviations: CC-case control; CL-chemiluminescence; CSS-cross-sectional; DR-diabetic retinopathy; ECL-electrochemiluminescence; HPLC-high performance liquid chromatography; LC-MS-high-sensitivity mass spectrometry; N/A-not available; RIA-radioimmunoassay; RCT-randomized controlled trial; T1-type 1; T2-type 2.

**Table 4:** Newcastle–Ottawa Scale for the assessment of case-control studies.

Study	Representativeness of Cases	Selection of Controls	Definition of Controls	Comparability of Cases and Controls	Exposure Ascertainment	Blinding of Outcome Assesement	Non-Response Rate	Outcome Assesement	Adequacy of Follow Up	Overall
Reddy et al. [27]	Moderate	Low	Low	Low	Low	Low/Moderate	Low	Low	Not applicable	Low/Moderate
Alcubierre et al. [15]	Moderate	Low	Low	Moderate	Low	Moderate	Low	Low	Not applicable	Low/Moderate
Castillo-Oti et al. [19]	Low	Moderate	Low	Low	Low	Unclear	Moderate	Low	Not applicable	Low/Moderate
Zahedi et al. [30]	Moderate	Low	Low	Low	Low	Low	Low	Moderate	Not applicable	Low

**Table 5:** Newcastle–Ottawa Scale for the assessment of cross-sectional studies.

Study	Representativeness of Sample	Non-Respondents	Ascertainment of Exposure	Comparability	Assesement of Outcome	Statistical Test	Overall
Almoosa et al. [16]	Low	High	Low	Moderate	Low	Low	Moderate
Bonakdaran et al. [18]	Moderate	Low/Moderate	Low	Moderate	Low	Low	Low/Moderate
Zhao et al. [31]	Moderate	Low/Moderate	Moderate	Moderate	Low	Moderate	Moderate
Girard et al. [21]	Moderate	Moderate	Moderate	Moderate	Low/Moderate	Moderate	Moderate
Xiao et al. [29]	Moderate	Low	Low	Moderate	Moderate	Low	Moderate
Chen et al. [20]	Moderate	Low	Low	Moderate	Moderate	Low	Moderate
Alam et al. [13]	Low	Low	Low	Low	Moderate	Low	Low/Moderate
Nadri et al. [26]	Low	Moderate	Low	Moderate	Low	Low	Low/Moderate
Long et al. [23]	Low	Moderate	Low	Moderate	Low	Low	Low/Moderate
Zoppini et al. [32]	Moderate	Low	Low	Low	Moderate	Low	Low/Moderate

**Table 6:** Newcastle–Ottawa Scale for the assessment of cohort studies.

Study	Representativeness	Selection	Ascertainment of Exposure	Comparability	Assessment of Outcome	Follow-Up	Adequacy	Overall
Kim et al. [8]	Moderate	Low	Low	Low	Low	Moderate	Low	Low/Moderate
Ashinne et al. [17]	Moderate	Low	Low	Low	Low	Moderate	Low	Low/MoDerate
Lopes et al. [24]	Moderate	Low	Moderate	Low	Moderate	Low	Low	Moderate
Usluogullari et al. [28]	Low	Low	Low	Low	Low	Not applicable	Low	Low
Millen et al. [25]	Low	Low	Low	Low	Low	Low	Low	Low

## Data Availability

Data extracted from included studies and data used for all analyses can be retrieved from the corresponding author.

## Supplementary Materials

The following supporting information can be downloaded at: https://www.mdpi.com/article/10.3390/biomedicines13010068/s1, Table S1: PRISMA checklist.
